# Health Promotion Education in India: Present Landscape and Future Vistas

**DOI:** 10.5539/gjhs.v4n4p159

**Published:** 2012-06-24

**Authors:** Sanghamitra Pati, Kavya Sharma, Sanjay Zodpey, Kavita Chauhan, Madhumita Dobe

**Affiliations:** 1 Indian Institute of Public Health, Bhubaneswar, India; 2 Public Health Foundation of India, New Delhi, India; 3 All India Institute of Hygiene and Public Health, Kolkata, India

**Keywords:** health promotion, health communication, public health education, India

## Abstract

‘Health promotion is the process of enabling people to increase control over and to improve their health’. This stream of public health is emerging as a critical domain within the realm of disease prevention. Over the last two decades, the curative model of health care has begun a subtle shift towards a participatory model of health promotion emphasizing upon practice of healthy lifestyles and creating healthy communities. Health promotion encompasses five key strategies with health communication and education as its cornerstones. Present study is an attempt to explore the current situation of health promotion education in India with an aim to provide a background for capacity building in health promotion. A systematic predefined method was adopted to collect and compile information on existing academic programs pertaining to health promotion and health education/communication. Results of the study reveal that currently health promotion education in India is fragmented and not uniform across institutes. It is yet to be recognized as a critical domain of public health education. Mostly teaching of health promotion is limited to health education and communication. There is a need for designing programmes for short-term and long-term capacity building, with focus on innovative methods and approaches. Public health institutes and associations could play a proactive role in designing and imparting academic programs on health promotion. Enhancing alliances with various institutes involved in health promotion activities and networking among public health and medical institutes as well as health services delivery systems would be more productive.

## 1. Introduction

“Health status of the population at large in India leaves much to be desired despite having a well-developed administrative system and technical skills” (WHO, 2007). Despite significant contributions towards the improvement of population health in last six decades, the health outcomes remain inadequate (John et al., 2011). The acceptance of this vast gap between its capabilities and the actual reality of the health situation in India has focused the attention of governmental and non-governmental agencies on rebuilding public health. Recently published Lancet Series on India is an attempt to describe the country’s evolving health fortunes in both encouraging and disturbing domain (Hortan & Das, 2011; Patel et al., 2011). The series of papers has highlighted the important health care challenges impeding our health system namely- co-existence of substantial burdens of infectious diseases, reproductive and child health problems, nutritional deficiencies, chronic diseases, and injuries (Paul et al., 2011). Several adverse social determinants along with behavioral risk factors like smoking, oral tobacco consumption, and alcohol abuse aggravate the situation (Patel et al., 2011). Health Promotion has emerged as a viable approach and tool for comprehensive and equitable health development globally and it holds considerable potential for strengthening public health in India (Riegelman & Garr, 2008).

Health Promotion can offer a public health practitioner the means by which health can be improved by enabling behavioral change that requires effort from the individual with support from and/or engaging with community-based interventions that enable the healthy choice to be the easy choice. The ideals of health promotion are based on the question “what cause health?” and what factors or determinants are linked to health and which of these are modifiable, and indeed how are they modifiable. This *salutogenic* approach to health is broader in perspective compared to the traditional preventive and curative approach (Antonovsky, 1996). The goal of health promotion is to empower individuals and communities to achieve the highest possible levels of well-being available to them. Common health promotion activities include adherence to healthy behaviors such as consumption of low-fat and low-cholesterol diets, regular exercise, not smoking and moderate consumption of alcohol (Wylie, 2004).

Research has shown that investment in public health is more efficient and effective than emphasis on curative and rehabilitative services. Based on this recognition and in light of perpetually limited public resources, many advanced economies are realigning public resources towards health promotion. However, there exists limited information on the management of this vital area of public health in low and middle income countries including India.

### 1.1 Defining Health Promotion

The underlying concept for modern health promotion can be traced to the World Health Organization’s Alma Alta declaration in 1964, namely, that ‘health is not merely the absence of disease but a state of complete physical, mental and social well being’ (WHO, 1946). Since then, health promotion has gradually become accepted as a vital and integral part of public health, and is now universally perceived as a holistic field of overlapping activity on primary, secondary and tertiary levels encompassing health education, lifestyle and preventative approaches. The first International conference on Health Promotion in Ottawa in 1986 presented a charter for action to achieve Health for all by the year 2000 and beyond (WHO, Ottawa Charter, 1986). Since then, concept of Health Promotion has come a long way with several Global conferences on Health Promotion taking place ever since, the sixth and the latest one was held at the Bangkok in 2005, the outcome of which is well known “Bangkok Charter for health promotion in a globalised world” (WHO, 2005). The definition of health promotion provided in the Ottawa Charter is the currently the most widely accepted one. In the Ottawa Charter, it is defined as: *“The process of enabling people to increase control over, and to improve, their health”*

There are five key strategies in the promotion of optimal health, as laid out in the Ottawa Charter. These are: building healthy public policy, creating supportive environments, strengthening community action, developing personal skills and reorienting health services. The World Health Organization (WHO) determined the principles for health promotion as (WHO, Bangkok Charter, 1986):

1) Population health focused
2) Social determinants focused
3) Able to combine a variety of methods
4) Building collaborative partnerships
5) Health workforce focused

### 1.2 Health Education and Communication in Health Promotion

In its endeavor to facilitate behavioral change for better health and making healthy choices the easy choices, health promotion activity heavily relies on health education and communication. Health education seeks to motivate an individual to accept a process of behavioral-change by directly influencing their value, belief and attitude systems, where it is deemed that the individual is particularly at risk or has been affected by illness/disease (WHO, Bangkok Charter, 1986). It includes strategies like behavioral change communication (BCC) and information, education and communication (IEC). Health communication encompasses the study and use of communication strategies to inform and influence individual and community decisions that enhance health. It links the domains of communication and health and is increasingly recognized as a necessary element of health promotion (Antonovsky, 1996). For individuals, effective health communication can help raise awareness of health risks and solutions provide the motivation and skills needed to reduce these risks, help them find support from other people in similar situations, and affect or reinforce attitudes. It can also increase demand for appropriate health services and decrease demand for inappropriate health services.

### 1.3 Health Promotion in India

India currently is faced with triple burden of diseases i.e. the existing communicable diseases, the emerging and re-emerging communicable diseases and the ever-increasing non communicable, lifestyle related diseases (WHO, Burden of diseases in India, 2005). All of these are one way or the other interlinked to lifestyles and behaviors at individual or community level. Behavioral change through effective communication has been perceived and proven as a useful and the most cost effective tool for addressing public health problems. Behavior in itself is multifactorial in causation and depends upon Healthy Public policy and supportive environment. This requires enabling health personnel to design suitable health promotional interventions to bring sustained change in behavior of the community (WHO, Burden of diseases in India, 2005). Recognizing the significant role of health promotion in spearheading public health interventions in India, it is imperative that health promotion should receive its deserving thrust. However, there is no systematic and comprehensive information available for existing teaching, training and capacity building activities pertaining to health promotion in India. In the light of above, a situational analysis was carried out to assess the current status of health promotion education across the country.

## 2. Methodology

The study involved a descriptive analysis of academic programs relating to health promotion. A systematic, predetermined strategy was undertaken for collecting and collating the gathered information. Data was compiled and assimilated into a matrix. The methodology used was similar to that adopted in an earlier study involving following process (Zodpey et al., 2009).

A thorough internet search was carried out to collect information in the public domain regarding courses offered in health promotion/health education/health communication in India. The search was conducted using search engines like Google, Dogpile etc. A set of key words was used for the search which included: health promotion, health communication, health education, public health, public health education, health behavior, social determinants of health, behavioral change communication, healthy community, and healthy lifestyle. The search was limited to courses offered in India and to collaborations between Indian and foreign institutes, if any. The websites of Association of Indian Universities (AIU), Universities Grants Commission (UGC), Medical Council of India (MCI), Nursing Council of India, Ministry of Health and Family Welfare (MOHFW) were also searched to find out courses offered in Health Promotion. A similar search was conducted through the websites of the Indira Gandhi National Open University (IGNOU), World Health Organization (WHO). The search was not restricted by course duration or the type of degree/certification awarded on successful completion. Detailed information about the courses was collected from the respective institutions or from the designated websites of these institutions. Short-term courses offered by various institutions, lasting from a few days to a few weeks, were disregarded and not taken into consideration. Informal discussions with students, faculty, and administrative staff of various medical and nursing colleges and public health institute were conducted to explore the existing options. Working professionals in the field of Public Health were also included in a formal discussion to identify their educational background and dimensions for the study. In situations where information was not updated on website, telephonic contacts were made with institutes and universities to procure detailed information. This study however did not review modules on development communication/health communication that are being delivered as part of journalism and mass communications programmes.

## 3. Results

Presently in India, selected organization/institutions are administering health promotion/health education as distinct educational program (Table 1). Currently Health Promotion teaching is being imparted as:

**Table 1:** Full time courses on health promotion/education

Institute	Course	University	Duration	Type of Course	Mode	Intake Capacity
Central Health Education Bureau	PG Diploma in Health Education	Delhi University	2 Year	Diploma	Contact	20
The Gandhigram Institute of Rural Health and Family Welfare Trust, Ambathurai	PG Diploma In Health Promotion and Education	Tamilnadu Dr MGR Medical University	1Year	Diploma	Contact	60
Public Health Foundation of India	Post Graduate Diploma in Health Promotion with applied focus on Tobacco Control	N/A	1 Year	Diploma	Online	40
All India Institute of Hygiene & Public Health, Kolkata	Diploma in Health Promotion & Education	West Bengal University of Health Sciences	1 Year	Diploma	Contact	60
Family Welfare Training & Research Center, Mumbai.	Diploma In Health Promotion Education	IIPS Mumbai	1 Year	Diploma	Contact	60
NIHFW	Certificate Course in Health Promotion	N/A	1 Year	Certificate	Distance Learning	N/A
IGNOU	Diploma in Nutrition and Health Education	N/A	1 Year	Diploma	Distance Learning	N/A
Open Universities	Diploma in Nutrition and Health Education	N/A	1 Year	Diploma	Distance Learning	N/A

1) Dedicated academic programs (full time/distance learning) focusing on health promotion
2) Health education with or without nutrition education, is imparted as an standalone study program (fulltime/distance learning)
3) Health Promotion, and/or health education and/or health communication are being taught as a module in Master of Public Health (MPH)/Diploma in Public Health (DPH)
4) Health Promotion and/or health education / communication are being imparted through Masters/Diploma in public health management courses.
5) Some elements of health promotion are being taught as an integrated manner in postgraduate and undergraduate medical education under community medicine.
6) Health promotion as a component in undergraduate allied health professionals (Nursing, physiotherapy and occupational therapy) curriculum and family medicine courses.

### 3.1 Dedicated Academic Programs in Health Promotion

Currently, exclusive study programs in health promotion are being offered by only three institutes. Family Welfare Training and Research Center (FWTRC), a central public health training institute in Mumbai offers diploma in health promotion education (DHPE) recognized by and affiliated to International Institute of populations Sciences (IIPS), Mumbai. The one-year course initially launched as diploma in health education (DHE) was later converted to health promotion keeping in view of the emerging need for teaching health promotion. The Gandhigram Institute of Rural Health and Family Welfare Trust(GIRHFW, 2011), Ambathurai in affiliation with Tamil Nadu Dr. M.G.R. Medical University is administering one-year program named post graduate diploma in health promotion and education (PGDPHE). It is a classroom based academic program and accepts students from social and health sciences disciplines having prior experience in community work. The one-year certificate course in health promotion is being offered by National Institute of Health and Family Welfare (NIHFW, 2011), an autonomous institute under the MOHFW, Government of India. This distance learning program adopts a combination of class room based contact session with field based learning. Certificate in health promotion program is one of its kinds to provide training in health promotion through distance education. Students graduating from these courses will work as health promotion experts with health system management unit, both central and state government, civil societies, multilateral agencies and public health institutes. Additionally they are employed as capacity building trainer for auxiliary health staff and health workers in health promotion. They also have opportunity to work as community health educators as well as school and workplace health promotion interventionist. Recently, government of India has launched national programme for control of Non-communicable disease, wherein these students can have important role in disease risk reduction and health promotion at all levels of healthcare delivery systems. Specialized opportunities in tobacco cessation, nutrition counseling, obesity prevention and health communication are also available for these students

### 3.2 Courses on Health Education/Communication

Exclusive programs on health education (postgraduate diploma / diploma) aimed towards producing a cadre of health educators for the community is presently being administered by All India Institute of Hygiene and Public Health (AIIH&PH, 2011), Kolkata and Central health education bureau (CHEB, 2011), New Delhi. AIIH&PH, an autonomous institute and one of the premier public health institutes of the country, offers one year diploma in health education while CHEB administers a two year post graduate diploma in health education (PGDHE) in affiliation with Delhi University.

Diploma in nutrition and health education (DNHE) is administered primarily through distance education mode by various open universities, the most important among them being Indira Gandhi National Open University (IGNOU, 2011). DNHE is a flexible study program with regular class room contact sessions. The study program includes a compulsory project work on health education.

### 3.3 Teaching of Health Promotion and Communication in Public Health Training

In India, currently public health training is primarily provided through master/diploma in public health, postgraduate/diploma in public health management, diploma/masters in health administration, diploma/master in health management.

Out of the currently undergoing master of public health (MPH) programs, only a selected few have a definite module on health promotion. The two-year MPH offered by Post Graduate Institute of Medical Research (PGIMER, 2011), Chandigarh offers an elective on health promotion. Tata Institute of Social Sciences (TISS), Mumbai is currently administering two public health related programs namely MPH and master of health administration (MHA) (TISS, 2011). Health education and communication are being taught under a single module in both these programs. The MPH program administered by Asian institute of public health, has a module on health behavior (AIPH, 2011). Although, the MPH program offered by Sree Chitra Tirunal Institute for Medical Sciences and Technology, Thiruvananthapuram does not include health promotion/education per se, some fundamental elements of the same are being covered. The MPH program of Birla Institute of Technology and Science has a module on communication in health care (BITS, 2011) and Jawaharlal Nehru University contains few components of health education as a part of community health nursing (JNU, 2011). The MHA program of All India Institute of Medical Sciences has a module on health communication applied to health care settings (AIIMS, 2011).

Postgraduate Diploma in Public Health Management is a one year course being administered by ten institutes namely AIIH & PH, Indian Institute of Public Health (IIPH) – Bhubaneswar, IIPH-Gandhi Nagar, IIPH-Hyderabad, NIHFW, SIHMC Gwalior, PGIMER, Chandigarh.(MOHFW, 2011) This course contains one credit based module on health promotion and communication.

### 3.4 Health Promotion Teaching in Medical Education

In India, every medical college has a department of community medicine as per MCI guidelines (MCI, 2011). These departments impart teaching and training at both undergraduate and postgraduate levels. There are around 184 medical colleges offering Doctor of Medicine (MD) in community medicine. National Institute of Health and Family Welfare (NIHFW), New Delhi offers MD in community health administration. Additionally, there are six medical colleges offering diploma in community medicine (DCM) accredited by MCI. As far as health promotion is concerned, the post-graduate teaching includes few elements of health education; healthy and harmful behavior, environmental hygiene, sanitation, tobacco control, nutrition, as a part of community medicine subject. In the undergraduate medical education, basic concepts of health education and hygiene behavior components are taught to the students. Diplomat of national board is administering DNB in family medicine which contains modules on health education and health communication as applied to family level health promotion. There are also few distance learning programs (PG Diploma) in family medicine which have a component on health education and counseling for healthy behavior.

Diploma in public health recognized by MCI is offered by medical colleges affiliated to universities. There are currently 39 medical colleges are administering DPH course (MCI, 2011). The DPH course includes a module on health education as applied to public health practice mainly focusing on hygiene and nutrition. Diploma in industrial health (DIH) accredited by MCI is being offered by three medical colleges (MCI, 2011). The course curriculum includes a module of health education.

### 3.5 Health Promotion / Education in Allied Health Profession Curriculum

There is a specialization of public health nursing in nursing stream which focuses public health as applied to nursing profession. Health promotion per se is not being included as a teaching module in nursing or occupational therapy/physiotherapy curricula (Indian Nursing Council, 2011)

## 4. Discussion

Present study involved conducting a situational overview of the existing system of health promotion education in India with an aim to provide critical inputs for health promotion planning and resource building. It is seen that, despite widespread recognition of the importance of health education, communication and promotion, at present, in India, there exists a clear cut deficiency in teaching of health promotion and communication across community medicine, public health and allied disciplines (Table 2). Currently, there are no structured and administered health promotion study programs in public health. Almost no formal organization of health promotion is found in the discipline of public health excepting few MPH programs focusing on health education and communication. Public health courses excepting PGDPHM don’t have health promotion and communication as a distinct module in their course program. Teaching of health promotion is still at the rudimentary level when compared to the public health education in western and high income countries.

**Table 2:** Overview of health promotion/education/communication teaching

University /Institute	Stand Alone	MPH/MHA	PGDPHM/PGDHHM	DNHE	DCM/DIH/DPH
JNU, Delhi		√^P^			
PGIMER, Chandigarh		√^P^			
TISS, Bombay		√^E^√^C^			
Sree Chitra Tirunal Institute for Medical Sciences & Technology. Trivandrum, Kerela.		√^P^			
Birla Institute of Science and Technology, Pilani		√^C^			
KLE University, Begaum		√p√^E^			
Interdisciplinary school of Health Sciences,Pune		√e√^C^			
School of Public health, SRM University		√p√^C^			
Allahabad Agricultural Institute		√p√c			
Asian Institute of Public Health (AIPH)		√^B^	√^B^		
IIPH- New Delhi			√^C^√^P^		
IIPH-Hyderabad			√^C^√^P^		
IIPH- Gandhi Nagar			√^C^√^P^		
IIPH- Bhubaneswar			√^C^√^P^		
SIHMC, Gwalior			√^C^√^P^		
AIIH&PH- Kolkata			√^C^√^P^		
MGIMS,Wardha			√^C^√^P^		
JIPMER,Puducherry			√^C^√^P^		
NIHFW, New Delhi	√^P^		√^C^√^P^		
Central Health Education bureau	√^E^				
The Gandhigram institute of rural health and family welfare trust, Ambathurai	√^P^√^E^				
All India institute of hygiene & public health, Kolkata	√^E^				
Family Welfare training & research center, Mumbai.	√^P^√^E^				
IGNOU				√^E^	
Kota University				√^E^	
UP Rajarshi Tandon Open University				√^E^	
Madhya Pradesh Bhoj(open) university				√^E^	
Karnataka State open University				√^E^	
Medical Colleges					√^E^

√^P^- Health Promotion √^E^- Health Education √^B^- Health Behavior √^C^- Health Communication

Health promotion content in medical and allied health professional curricula is yet to gain its due coverage. During the doctorate in medicine and in community medicine, elements of health promotion are covered but not in a systematic way. Traditional approaches of health education are being emphasized as the tools for behavioral change thus undermining the importance of health communication. Present MD (Community Medicine) curricula are yet to infuse the holistic concept and multidisciplinary approach of health promotion. Conceptual understanding of health promotion needs to be integrated into undergraduate medical and allied health professional education.

Clearly defined content of health promotion has to be incorporated and made an integral component of public health educational programs. Many MPH courses do not have any compulsory module on health promotion/communication. This could reduce the scope of having adequate human resources trained in health promotion. Master of public health programs with specialized tracks in health promotion could be one of the prime objectives which in turn would encourage formation of public health professional trained in health promotion and thus pave path for a cadre health promotion workers in the country. Coordination between institutes offering courses on health promotion, health education and public health is to be geared to ensure a climate of health promotion.

Full-time study programs on health promotion are few which is of concern. When compared to western public health systems, India is lagging behind in terms of health promotion education. There are around 205 Universities and Colleges in USA administering full time Postgraduate and Graduate Health Promotion courses and 46 Universities and Colleges offer distance/online Postgraduate /Graduate Health Promotion courses. There are 191 institutions in UK which impart full-time Health Promotion training courses and 328 institutions and universities in mainland Europe offering full-time Health Promotion (Pringle et al., 1997; Toon et al., 1998).

Presently there is no uniformity in curricular content amongst institutes offering exclusive health promotion programs. Existing health promotion programs need to be reviewed for their curricular homogeneity with necessary modifications. There is also need for sharing of knowledge and skills, best practices and evidence based health promotion interventions across public health and medical institutes imparting health promotion/education programs.

Health communication, a key strategy of health promotion, holds tremendous importance in a heavily populated country like India with more than one billion population. At present, there are very few programs in public health which encompass health communication in their courses. Health promotion-communication teaching could foster the students with requisite skills and knowledge in creating awareness of health and nutrition issues, change norms and practices, and persuade individuals, families, and communities to make positive behavior changes.

Presently India, a country under rapid demographic and socioeconomic transition is experiencing triple burden of diseases, which include infectious, reemerging and chronic diseases. All these involve health promotion at the very core of their interventions. Tertiary and secondary care is concerned with curative services while promotive services ought to be delivered at every level. Particularly, challenges like coexistent over and under nutrition, multiple concurrent chronic conditions (diabetes and TB), early onset of cardio-metabolic risk factors, tobacco and alcohol abuse, road traffic injuries require adoption of a holistic perspective rather than singular disease specific approach. Health promotion through its integral behavioral change intervention aimed towrdas improving nutrition, life style and health behavior could enable every person to achieve optimal health related quality of life.

Various actors that would play a determining role in health promotion training and practice range from NGOs, academic institutes, research and development organizations, international donor agencies, public health associations, medical and allied health professionals, social scientists and workers. Health promotion practice by each and every actor within the health system and its overlapping domains is crucial. Such endeavor would necessitate a continuous need-based supply of health promotion specialists into the public health arena. At this stage, it might be difficult to predict the total volume of health promotion personnel who would effectively be able to meet the needs. Present study has not estimated the requisite number of health promotions professionals to deliver the services across all cadres and other health related sectors.

As India is new entrant into this health promotion movement and we are still in the beginning of health promotion education and capacity building, further exploratory studies elucidating the needs and resources as well as the current practice and level of health promotion need to be undertaken. In this effort, present study is the first attempt to bring health promotion to the forefront of attention and advocate towards energizing the process of health promotion education, training and practice in the country.

## 5. Conclusion

Health promotion is a diverse and growing field of public health. While education in health promotion is vital, there needs to be a strong emphasis on strengthening health promotion practice and education, with special focus on using innovative and attractive formats. Particularly, in India, there is a need to broaden the scope of public health education to include health promotion teaching as essential component. Enhancing alliances with various institutes involved in health promotion activities and networking among public health and medical institutes and health services delivery systems would be more productive. An important challenge is to link health promotion teaching with national public health goals and local public health problems. Greater integration of health promotion with undergraduate medical and allied health disciplines is needed. Setting based (school, workplace, community, health care) health promotion and community-academic partnerships hold the key for addressing the problem at multiple levels. People working in these settings could be trained strategically in health promotion so as to deliver the needful services and thus contribute effectively.
